# The Structural Evolution of *β*-to-*α* Phase Transition in the Annealing Process of Poly(3-hydroxybutyrate-co-3-hydroxyvalerate)

**DOI:** 10.3390/polym15081921

**Published:** 2023-04-18

**Authors:** Jian Yang, Xianggui Liu, Jinxing Zhao, Xuelian Pu, Zetong Shen, Weiyi Xu, Yuejun Liu

**Affiliations:** 1 Key Laboratory of Advanced Packaging Materials and Technology of Hunan Province, Hunan University of Technology, Zhuzhou 412007, China; jianyang@hut.edu.cn (J.Y.); x15630499835@163.com (J.Z.); abdabd534@163.com (X.P.); ttsz966@sina.com (Z.S.); xuweiyi2002@163.com (W.X.); 2 National & Local Joint Engineering Research Center for Advanced Packaging Material and Technology, Hunan University of Technology, Zhuzhou 412007, China; 3 Guangdong Huatong New Material Technology Co., Ltd., Dongguan 523591, China; xgliu507@outlook.com

**Keywords:** poly(3-hydroxybutyrate-co-3-hydroxyvalerate) (PHBV), annealing, *β*-form, microstructure

## Abstract

In this study, the structural and property changes induced in the highly ordered structure of preoriented poly(3-hydroxybutyrate-co-3-hydroxyvalerate) PHBV films containing the *β-*form during annealing were investigated. The transformation of the *β-*form was investigated by means of in situ wide-angle X-ray diffraction (WAXD) using synchrotron X-rays. The comparison of PHBV films with the *β-*form before and after annealing was performed using small-angle X-ray scattering (SAXS), scanning electron microscopy (SEM) and differential scanning calorimetry (DSC). The evolution mechanism of *β-*crystal transformation was elucidated. It was revealed that most of the highly oriented *β-*form directly transforms into the highly oriented *α-*form, and there might be two kinds of transformations: (1) The *β*-crystalline bundles may be transformed one by one rather than one part by one part during annealing before a certain annealing time. (2) The *β*-crystalline bundles crack or the molecular chains of the *β-*form are separated from the lateral side after annealing after a certain annealing time. A model to describe the microstructural evolution of the ordered structure during annealing was established based on the results obtained.

## 1. Introduction

Traditional fossil plastics used as short-life-cycle products such as packaging have provided consumers with great convenience, but these degradation-resistant plastics are continuously accumulating in the environment, leading to ecological problems, including solid waste disposal problems and marine pollution, among others. Therefore, the research and development of biodegradable polymer-based solutions for short-life-cycle products has become a key issue in recent years [1]. Polyhydroxyalkanoates (PHAs) are competitive biodegradable resins that can be isolated from bacteria through biological fermentation [2]. Among various PHAs, poly(3-hydroxybutyrate-co-3-hydroxyvalerate) (PHBV) was the first commercialized bacterial copolyester [3,4] and has been studied for more than three decades. However, the mechanical properties of PHBV deteriorate due to secondary crystallization [5,6]. The size of the spherulites increases during room-temperature storage, which can lead to the detrimental embrittlement of the material [5,6]. Studying the crystal structure is essential for establishing the relationship between the structure and physical properties of these polymers. According to research, there are two crystal modifications in PHBV (the *α-*form and the *β-*form). The *α-*form is commonly found in cooling melts, and the crystallographic data, lattice parameters and molecular conformation of the *α*-crystal have been reported in several works [7,8,9,10], as shown in Table 1. Yokouchi et al. [8] first discovered the *β-*form in uniaxial cold-drawn PHB (poly(3-hydroxybutyrate), one of the PHAs) films. Iwata et al. [11,12,13,14,15,16] prepared PHBV films and fibers with high mechanical properties. They proposed that the existence of *β-*form crystals was one of the important factors responsible for the enhancement of mechanical properties [11] and the prevention of performance degradation [16]. Yamane et al. [17,18] also found that the toughness of PHB increased with the increasing proportion of the *β*-form and the orientation of the *α*-form. In our previous work [19], a mechanism to explain the role of the *β-*crystal in keeping the ordered microstructures stable and enhancing the mechanical properties was proposed. As reported, the *β-*form adopts a planar zigzag conformation [20,21]. The crystallographic data, lattice parameters and molecular conformation for *β*-crystals are compared in Table 1. However, some researchers argue that the *β-*form is a mesophase that consists of highly oriented and stretched molecular chains ordered in one direction [22,23].

In addition, the formation mechanism of *β*-crystals is controversial. The first proposed mechanism suggests that the *β-*form comes from the amorphous structure between the lamellae of the *α-*form [8,21,25,26,27,28]. The second one proposes that the *β-*form comes from the phase transition of the *α-*form [24]. Meanwhile, Xia et al. [29] proposed that *β-*crystals originate from both *α-*crystals and amorphous regions, and the proportion of *β* crystals is different in different tensile stages. Phongtamrug et al. [24] studied the formation process of the *β*-crystal of PHB in detail. There are long or short tie molecules between the lamellae of the *α*-form. During the stretching process, the short tie molecules are under high tension, and the region connected with the tie molecules of crystalline lamellae of the *α*-form with short tie molecules are stretched into *β*-crystals together. The arrangement of *α* lamellae becomes loose, and the orientation degree decreases. They also found that the lamellar thickness of *α*-crystals is ca. 50 Å in the tensile direction and ca. 270 Å in the lateral direction, while *β*-crystals are much smaller, with a size of ca. 90 Å in the tensile direction and ca. 40 Å in the lateral direction. Xia et al. [29] studied the *β*-crystal formation mechanism during the stretching of poly(3-hydroxybutyrate-*co*-4-hydroxybutyrate) (P3HB4HB) (4HB: 15 mol%), and the conclusion was similar to that of Phongtamrug et al., suggesting that the *β*-crystal formation mechanism may be common for PHAs with 3HB as the main monomer.

However, the transformation process of the *β-*form is still unclear and has been relatively less studied. Murakami et al. [30] studied the *β-*crystal transition during the heating process using Raman spectroscopy and found five characteristic spectra related to the *β-*form, which disappeared above 130 °C, indicating that the transition temperature of the *β-*form is approximately 130 °C. Kabe et al. [31] studied the transformation process of the *β-*form during the process of heating, and the results of fast differential scanning calorimetry showed that the sample containing the *β-*form had a small exothermic peak in the range of 110–140 °C compared with the sample without *β-*crystals, which may correspond to the transition of the *β-*form. The results of wide-angle X-ray diffraction (WAXD) indicated that the *β-*form may be transformed into *α-*crystals or amorphous structures in the range of 90–130 °C. Yamane et al. [32] studied the structure and properties of PHB fibers containing *β*-crystals after annealing for a certain time at different temperatures and tensions. When the tension is less than 50 MPa, the diffraction intensity of the *β-*crystal decreases with the increase in temperature. However, when the tension is higher than 100 MPa, the diffraction intensity of the *β-*crystal increases with the increase in temperature (not higher than 125 °C). This indicates that the temperature and tension are two critical factors that influence the transformation of *β*-crystals.

Thermal annealing treatment is an important process for adjusting the structures of semicrystalline polymers, including the relaxation of oriented chains and the rearrangement of chain segments [33]. For instance, injection-molded parts always need annealing to regulate their microstructure and improve their impact toughness [34,35]. Annealing is also one of the key steps in the spinning process, by which the structures of the synthetic fibers reach a new thermodynamic equilibrium, and results in improved mechanical properties and dimensional stability [36]. PHBV is one of the most important members of the PHA family, and although some works have studied the transformation process of the *β-*form during the process of heating, the transformation process during annealing is still ambiguous. In order to gain further insights into the structural evolution of oriented PHBV films with *β*-crystals during annealing, we studied the process by in situ WAXD using synchrotron X-rays, together with small-angle X-ray scattering (SAXS), scanning electron microscopy (SEM), etc. In the first step, two-step-drawn PHBV films were prepared, and the structural evolution during annealing was observed. Subsequently, the structural and property differences between annealed and unannealed PHBV films were comparatively investigated. Finally, the evolution mechanism of *β-*crystal transformation was proposed. The clarification of chain disentangling and the aggregation structure in *β-*form crystallites may be an important point for clarifying the essential features of PHBV and its family.

## 2. Materials and Methods

### 2.1. Materials and Sample Preparation

PHBV powder was obtained from TianAn Biologic Materials Co., Ltd., Ningbo, China. The molecular weight of PHBV was *M_w_* = 471,400 g/mol, and *M_n_* = 196,400 g/mol. The 3-hydroxyvalerate (HV) ratio in PHBV was 1 mol%. The glass transition temperature of PHBV is 0 °C, and the melting temperature is 165 °C. The material was dried for 48 h in a 60 °C vacuum drying oven before use.

**Figure 1:**
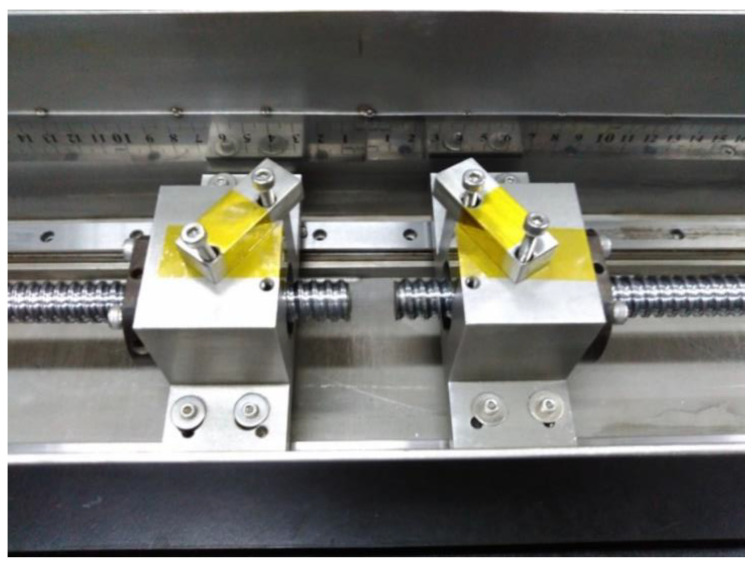
Part of the homemade stretching machine with a water channel.

The fully dried PHBV powder was heated at 200 °C for 3.5 min to melt it, pressed into a film and then immediately quenched in ice water. The quenched film was directly cut into rectangular films and stretched to ten times the initial length in a homemade stretching apparatus (Figure 1) at 0 °C. After fixing the length at room temperature for about 15 min in case of retraction, the films were taken out from the equipment and cut into films with a length of 30 mm. All the films (approximately 30 × 4 × 0.08 mm^3^) were stored at −20 °C before use.

**Scheme 1:**
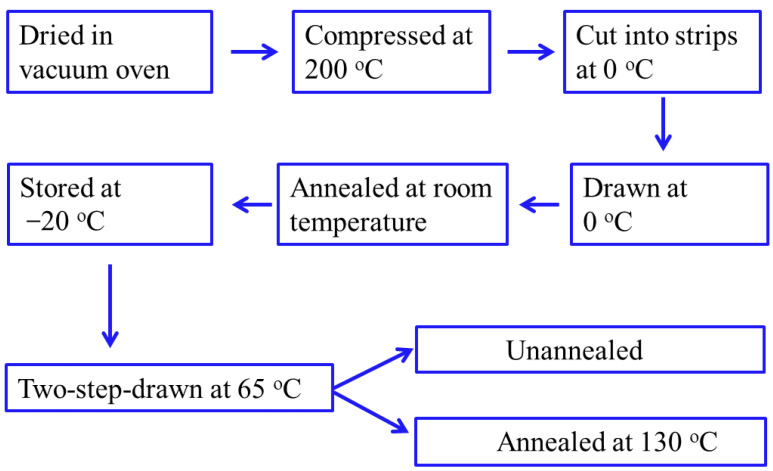
Experimental routes to prepare unannealed and annealed samples.

The films were stretched to 150% (rate: 50 μm/s; temperature: 65 °C) and kept under tension at 65 °C for 5 min in the Linkam TST350 hot stage. The *β*-crystals formed mainly from the drawing at 65 °C, as we proved recently [37]. After storing the samples at room temperature for more than 24 h, the two-step-drawn films (unannealed samples) were prepared. The two-step-drawn sample was heated to 130 °C at a rate of 100 °C/min with a fixed length in the hot stage and annealed at 130 °C for 45 min. After storing the samples at room temperature for more than 24 h, the annealed sample was prepared. The experimental routes are illustrated in Scheme 1. WAXD data were collected in the process of annealing at 130 °C for 45 min.

### 2.2. Characterization

In situ WAXD measurements were taken at the beam Line 1 W2A-SAXS in the Beijing Synchrotron Radiation Facility [32]. Scattering patterns were collected by a MAR CCD (MAR-USA) detector with a resolution of 2048 × 2048 pixels, and each pixel size was 79 × 79 μm^2^. The sample-to-detector distance was 150.7 mm, and the wavelength of the radiation source was 1.54 Å. The image acquisition time was 20 s. All X-ray images were corrected for air fluctuations and background scattering.

Two-dimensional SAXS diagrams of the two-step-drawn PHBV films were generated using a Xeuss 2.0 SAXS/WAXD System (France). The incident X-ray beam was Cu K*α* with a wavelength of 1.54 Å. Each data frame was collected with an acquisition time of 900 s.

The melting behavior of the films (annealed and unannealed) was examined by a TA DSC-Q2000. The reported melting temperature was from the first heating from −10 °C to 200 °C at a rate of 10 °C/min under nitrogen. Before measurements, the instrument was calibrated with indium.

To observe the morphology of the fractured surface along the stretching direction, the films were torn apart in liquid nitrogen, followed by surface etching. The surface etching operation was described in our previous work [19]. After a thin conductive platinum layer was sputter-coated onto the samples, a HITACHI SU8020 scanning electron microscope was utilized to obtain high-resolution images of the fractured surfaces with an accelerating voltage of 3 kV.

In order to study the mechanical properties of the annealed and unannealed films, stress–strain curves were recorded in the Linkam TST350 hot stage at room temperature.

## 3. Results and Discussion

### 3.1. Microstructural Evolution of PHBV Film with β-Form during Annealing

**Figure 2:**
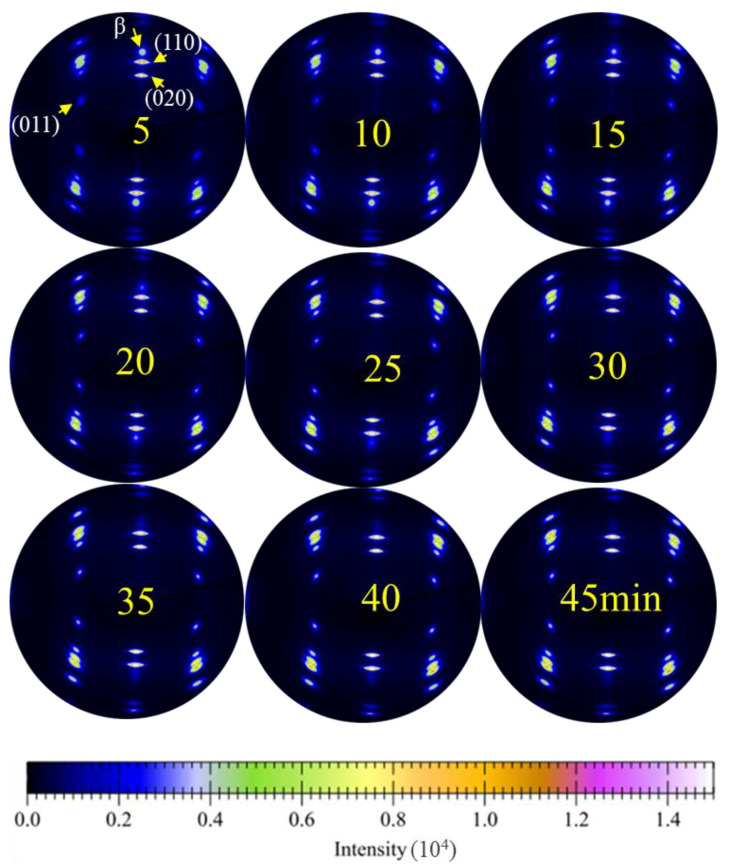
Real-time 2D-WAXD patterns of the two-step-drawn PHBV films obtained during annealing at 130 °C at different times, as labeled on the patterns. The drawing direction is horizontal and is defined as the meridian in this paper.

To trace the structural evolution of the PHBV film with *β-*crystals during annealing with the length unchanged, 2D-WAXD was used to study the annealing process at 130 °C. The crystallinity, relative content of *β-*crystal (R*_β_*_/(020)_), degree of orientation, crystallite size and *d*-spacing were studied. Typical 2D-WAXD diagrams of two-step-drawn PHBV films during annealing at 130 °C at different times are shown in Figure 2. As indicated in the image, the diffraction from the *β-*crystal is broad but spotlike along the equator, suggesting highly oriented crystallites with small sizes, and molecular chains of the *β-*crystal are packed roughly along the drawing direction [15]. The intensity of diffraction spots along the equator of the *β-*crystal decreases with the annealing time and disappears when the drawn film is annealed for more than 42 min, suggesting that the shrinkage and reorganization of molecular chains of the *β-*crystal occur after annealing.

**Figure 3:**
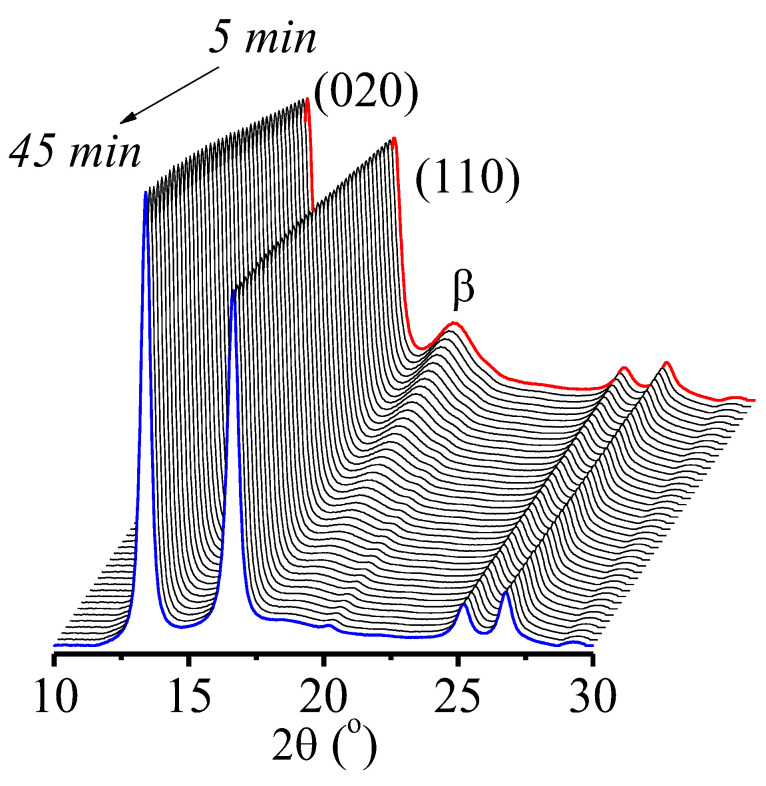
Equatorial line of the WAXD pattern of the two-step-drawn PHBV film observed during annealing at 130 °C.

Figure 3 shows the 1D WAXD intensity profiles of the two-step-drawn PHBV film as a function of annealing time. The diffractions along the equator in the 2D WAXD diffraction patterns of the two-step-drawn PHBV film were converted into 1D equatorial profiles by circular averaging. The three diffraction peaks, 13.7°, 17.1° and 19.7°, correspond to (020)_α_, (110)_α_ and (110)*_β_* [24], respectively, which are indicated by arrows in Figure 2. It is noted that the intensity of the *β-*form diffraction peak decreases, whereas the intensities of the two *α-*form diffraction peaks increase with increasing annealing time.

**Figure 4:**
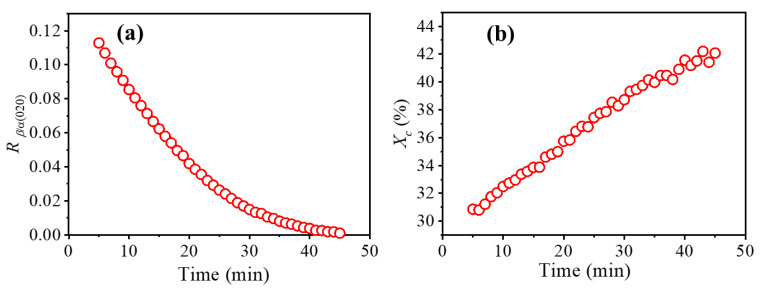
(**a**) *R_β_*_/(020)_ and (**b**) *X_c_* of two-step-drawn PHBV films during annealing at 130 °C.

**Figure 5:**
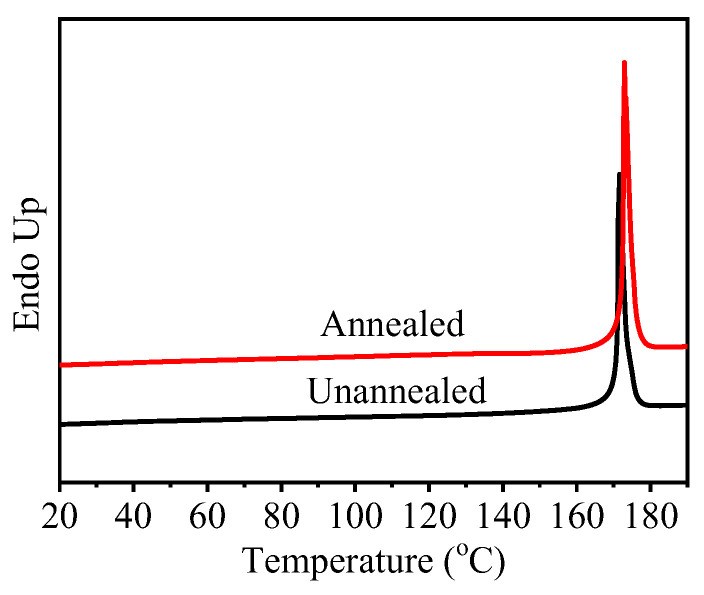
The first DSC melting curves of the unannealed and annealed films.

The relative content of *β-*crystals (*R_β/_*_(020)_) and crystallinity (*X_c_*) of the two-step-drawn PHBV film as a function of annealing time are shown in Figure 4. The relative content of *β-*crystals *R_β/_*_(020)_ was calculated from the integrated intensity of the reflection intensity ratio between the *α-* and *β-*crystals in 2D-WAXD, for which the diffraction peak (020)*_α_* and the *β-*form were used [16]. *X_c_* was calculated according to the work of Chen et al. [38]. *R_β/_*_(020)_ decreases and the crystallinity increases with increasing annealing time. These results suggest that the *β-*form and the amorphous phase transform into the *α*-form during the annealing process. Figure 5 shows the DSC heating curves of the unannealed sample and the annealed sample. The melting temperature of the annealed sample is 172.9 °C, which is a little higher than the melting temperature of the unannealed sample (171.6 °C), suggesting that the lamellae in the unannealed sample are thinner [39,40]. The melting enthalpy of the annealed sample (93.8 J/g) is higher than that of the unannealed sample (87.3 J/g), which is in accord with the WAXD results. It should be noted that the *β-*crystal in the unannealed sample transforms into an *α-*crystal in the process of elevating the temperature before melting, and here, the enthalpy of the unannealed sample should be larger than its original enthalpy. Even so, the measured enthalpy of the annealed sample is still higher than that of the unannealed sample.

**Figure 6:**
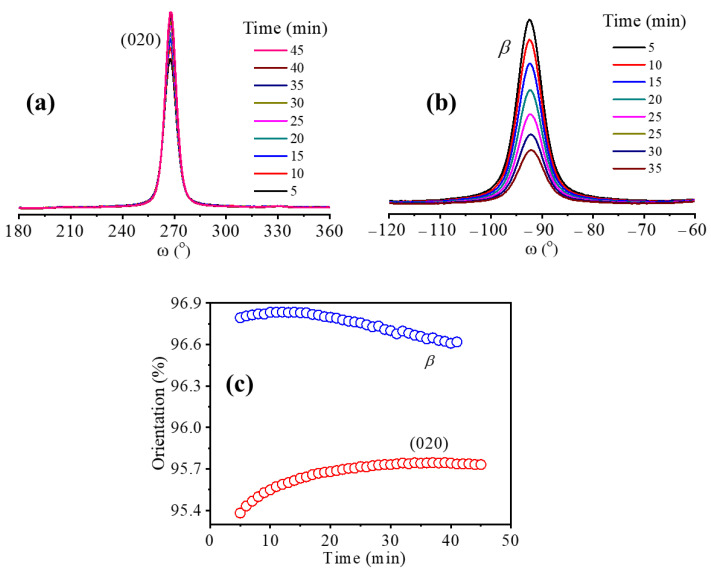
(**a**) Azimuthal intensity distribution of (020)*_α_* reflection and (**b**) *β* reflection; (**c**) changes in orientation of (020) and *β* reflections of two-step-drawn PHBV films during annealing at 130 °C.

As shown in Figure 6a,b, the intensity distribution along the azimuthal angle ω is plotted for (020)*_α_* and *β*, which changed with the annealing time. With increasing annealing time, the ω-scanned peak profile of the *β-*form became broader, indicating a lower degree of *β-*form orientation, while the orientation of the *α-*form showed the opposite trend. The orientations of *α*- and *β-*forms were calculated from the full width at half-maximum (FWHM) of the diffraction peaks along ω [41], and the orientations at different annealing times are summarized in the plot in Figure 6c. This suggests that the *β-*form with high orientation might directly transform into the *α-*form. The crystallite orientations of the *β-*form gradually decreased from 96.8% to 96.6% during annealing, indicating that annealing occurs with the tilting or deformation of *β-*form crystallites, and the stacking model of molecular chains in *β-*form crystallites, both in the stretching direction and perpendicular to the stretching direction, may be disarranged and transform into *α-*crystal chains.

**Figure 7:**
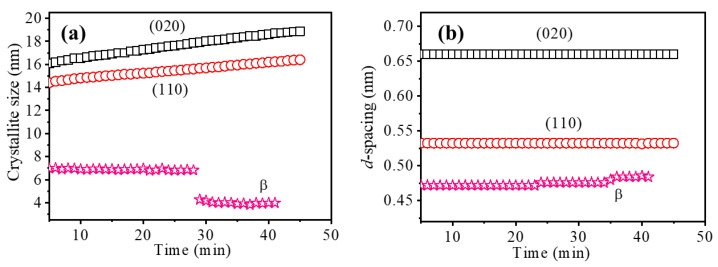
Changes in (**a**) crystallite size and (**b**) *d-*spacing of the (020), (110) and *β* reflections of two-step-drawn PHBV films during annealing at 130 °C.

The crystallite size was calculated using the Scherrer equation [42], and the *d-*spacing was determined by using the Bragg equation [15], as shown in Figure 7. The crystallite sizes of the *α-*form gradually increased during annealing, suggesting that molecular chains were folded and packed in the lateral direction. Meanwhile, the *d-*spacing of (020)*_α_* and (110)_*α*_ remained constant, suggesting that the *α-*crystal is very stable at the annealing temperature. Then, however, the crystallite sizes of the *β-*form decreased abruptly from 6.97 nm to 3.98 nm, and the *d-*spacing increased from 0.471 to 0.485 nm during the process of annealing, suggesting that the molecular chains in *β-*form crystallites were disentangled, including molecular chain segment movement, rearrangement and bond angle changes, and folded and packed into the *α-*form or released from the crystallite lateral to the amorphous region in the process of annealing.

An interesting point to note is that the orientation and relative content of *β-*form crystallites decreased and *d-*spacing increased gradually in the annealing process, whereas the crystallite size of the *β-*crystal decreased abruptly when the annealing time was 27 min. A possible explanation for this interesting phenomenon will be discussed in the next section.

### 3.2. The Comparison of PHBV Films with β-Form before and after Annealing

**Figure 8:**
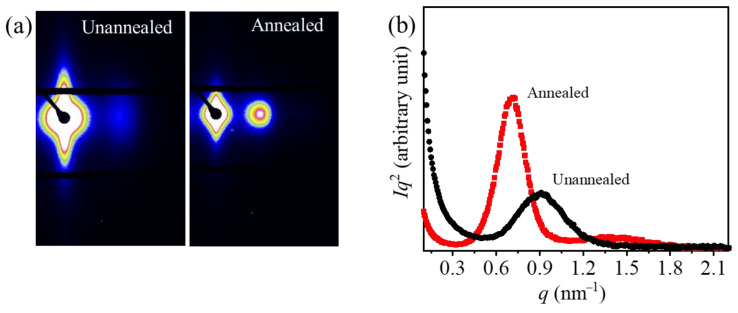
(**a**) SAXS patterns of unannealed and annealed films. The drawing direction is horizontal and is defined as the meridian. (**b**) Meridional lines of Lorentz-corrected 1D SAXS of unannealed and annealed samples.

**Figure 9:**
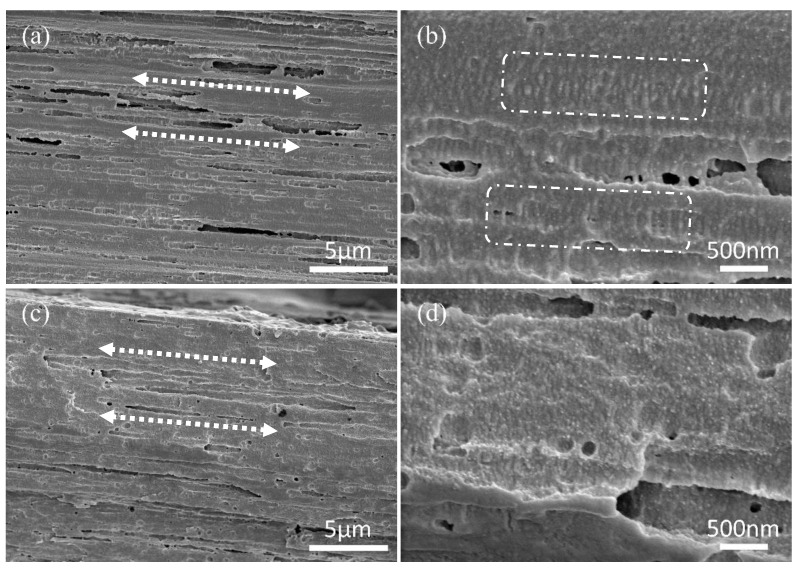
SEM images of the fractured surface of unannealed (**a**,**b**) and annealed (**c**,**d**) samples torn apart along the drawing direction in low-temperature medium.

Once we are aware of the structures of PHBV films with the *β-*form before and after annealing, we can better understand the structural transformation of the *β-*form. As shown in Figure 8, SAXS measurements were taken to detect the evolution of the lamellar stacking model of PHBV with the *β-*form before and after annealing. The SAXS patterns of annealed and unannealed samples show point scattering in the meridional direction and streak scattering along the equator, indicating the existence of shish-kebab structures [15,18,27]. The equatorial streaks across the beam stop in both SAXS patterns indicate the existence of fibrils (shish), while the spots along the meridional direction are ascribed to the oriented lamellar structures (kebab) [19,43,44,45]. The morphologies of the fractured surface of annealed and unannealed samples are observed in scanning electron microscopy images (Figure 8), in which highly oriented thread-like shish and kebab structures are marked, which is in accordance with the data from SAXS. The shish kebabs are arranged along the stretching direction and indicated by the white dotted arrows, and the kebabs are marked by the white dashed rectangle. The kebabs cannot be easily distinguished in the picture of the annealed sample (Figure 9d), because the crystallinity of the annealed sample is high, and little amorphous material was removed by surface etching. Comparing the annealed and unannealed samples, it is clear that the scattering intensity of the annealed sample is stronger than that of the unannealed sample. This suggests that there is an increase in the electron density between the crystalline lamellae and the amorphous layers after annealing. This is attributed to the existence of the *β-*form located between the crystalline lamellae of the *α-*form, and the density difference between the *α-*form (1.28 g/cm^3^) and *β-*form (1.25 g/cm^3^) crystals is insignificant in the unannealed film [24]. The one-dimensional scattering intensity distributions derived from Lorentz-corrected SAXS patterns along the stretching direction are plotted in Figure 8b. The peak position of the SAXS pattern of the unannealed film moves to a lower *q* value after annealing, which suggests there is growth in the long period (*Lp*). The *Lp* of the stacked lamella for the unannealed sample calculated by Bragg’s law is ca. 6.9 nm, and it increases to 8.8 nm after annealing.

**Figure 10:**
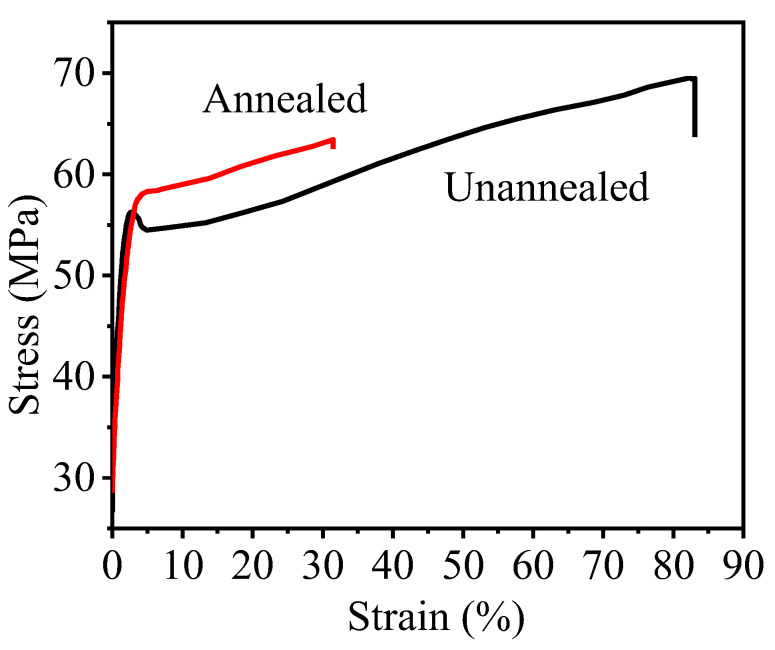
Engineering stress–strain curves of unannealed and annealed films.

As shown in Figure 10, the unannealed film presents better mechanical properties compared with the annealed sample. The unannealed sample with *β-*crystals shows better ductility and strength, which is consistent with previous studies [19,46].

### 3.3. Phase Transition Mechanism of β-Form during Annealing

**Figure 11:**
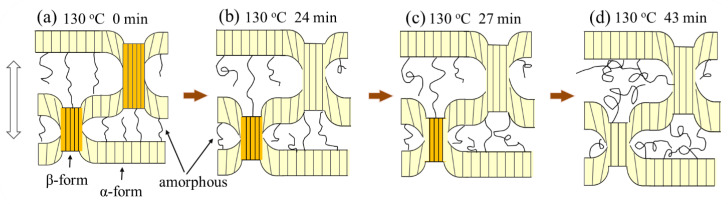
The model illustrates the higher-order structural evolution of the preoriented PHBV film during annealing. The vertical arrow indicates the oriented direction.

In this subsection, we try to describe the phase transformation mechanism of the *β-*crystal during annealing. According to the conclusions mentioned above, a new structural evolution behavior model in the process of annealing is proposed, as shown in Figure 11.

(a) Oriented lamellar crystals of the *α-*form are aligned by the previous stretching process, and *β-*crystalline bundles are generated from the tie chains between lamellar crystals of the *α-*form. The structure model proposed here is also reported in our previous work [19] and other studies [24,31].

(b) Most of the highly oriented *β-*form directly transforms into the highly oriented *α-*form, which can be concluded from the decrease in the relative content of the *β-*form and the increase in crystallite size and the orientation of the *α-*form in this annealing process. The *β*-crystalline bundles may be transformed one by one rather than one part by one part because the crystallite sizes of the *β*-form remain constant before 27 min. “One by one” means that the whole individual *β*-crystalline bundle transforms instantaneously, while “one part by one part” means that the individual *β*-crystalline bundle gradually collapses.

(c) The *β*-crystalline bundles crack or the molecular chains of the *β-*form are separated from the lateral side at an annealing time of 27 min, which can be concluded from the crystallite sizes of the *β-*form abruptly decreasing by nearly half, before which the *d*-spacing of the *β-*form increases slightly after an annealing time of 24 min.

(d) *β-*Crystals transform into *α-*crystals or amorphous chains completely, and the *Lp* of the stacked lamellae and the crystallite size of the *α-*form increase steadily throughout the whole annealing process.

We recently studied the transformation process of the *β*-form crystals in PHBV during the heating process from 65 °C to 145 °C at 2 °C/min, and this work has been accepted by the Chinese journal *Polymer Bulletin*, which will be published soon [37]. By comparison, the transformation processes of *β-*form crystals during annealing and heating are similar. The difference between them is that there is a clear intermediate state in the transformation process during annealing. The crystallite sizes of the *β*-form remained constant before 27 min and decreased abruptly by half, while the crystallite sizes of the *β*-form remained constant during nearly the whole heating process until vanishing. The *β*-crystalline bundles cracked or the molecular chains of the *β*-form were separated from the lateral side during annealing, and then the crystallite sizes decreased by half and were constant for more than 10 min. It is widely known that there is an enormous impact of annealing on melt-spun PHA fibers [47]. The model of the phase transformation mechanism of the *β-*crystal during annealing we propose here may provide guidance on thermal annealing treatment. According to the model, it is possible to control the crystallite size and content of *β-*crystals by setting the appropriate annealing time.

## 4. Conclusions

Time-resolved WAXD of two-step-drawn PHBV films was investigated during annealing at 130 °C by using synchrotron radiation, together with the measurement of DSC, SAXS, SEM and stress–strain curves. It was revealed that most of the highly oriented *β-*crystals directly transform into the highly oriented *α-*form, and there might be two different kinds of transformation forms of the *β-*crystal. First, the *β*-crystalline bundles may be transformed one by one rather than one part by one part because the crystallite sizes of the *β*-form remain constant during annealing before a certain time. Second, the *β*-crystalline bundles crack or the molecular chains of the *β-*form are separated from the lateral side after annealing for a certain time, which can be concluded from the crystallite sizes of the *β-*form abruptly decreasing by nearly half, and the *d*-spacing of the *β-*form increases slightly. This study has shed new light on the transformation mechanism of the *β-*form of PHBV during the annealing process and may provide guidance on the thermal annealing treatment of PHAs with *β-*crystals.

## Figures and Tables

**Table 1:** Crystallographic data, lattice parameters and molecular conformation for the *α*- and *β*-crystals of PHB.

	Lattice	Space Group	*a* (Å)	*b* (Å)	*c* (Å)	Molecular Conformation	Reference
*α-*form	Orthorhombic	*P*2_1_2_1_2_1_^-^D_2_^4^	5.67	13.15	5.91	2_1_ helix	Wang et al. [7]
*β-*form	Hexagonal	*P*3_2_21	9.22	9.22	4.66	All-trans zigzag	Phongtamrug et al. [24]

## Data Availability

Not applicable.
